# The effect of certification and accreditation on quality management in 4 clinical services in 73 European hospitals

**DOI:** 10.1093/intqhc/mzu023

**Published:** 2014-03-09

**Authors:** Charles D. Shaw, Oliver Groene, Daan Botje, Rosa Sunol, Basia Kutryba, Niek Klazinga, Charles Bruneau, Antje Hammer, Aolin Wang, Onyebuchi A. Arah, Cordula Wagner, on behalf of the DUQuE Project Consortium, N Klazinga, DS Kringos, K Lombarts, T Plochg, MA Lopez, M Secanell, R Sunol, P Vallejo, P Bartels, S Kristensen, P Michel, F Saillour-Glenisson, F Vlcek, M Car, S Jones, E Klaus, P Garel, K Hanslik, M Saluvan, C Bruneau, A Depaigne-Loth, C Shaw, A Hammer, O Ommen, H Pfaff, O Groene, D Botje, C Wagner, H Kutaj-Wasikowska, B Kutryba, A Escoval, M Franca, F Almeman, H Kus, K Ozturk, R Mannion, OA Arah, A Chow, M DerSarkissian, C Thompson, A Wang, A Thompson

**Affiliations:** 1 Centre for Clinical Governance Research, University of New South Wales, Sydney, Australia; 2 London School of Hygiene and Tropical Medicine, London, UK; 3 NIVEL, Netherlands Institute for Health Services Research, Utrecht, the Netherlands; 4 Avedis Donabedian Institute, Universitat Autonoma de Barcelona, Catalonia, Spain; 5 Red de Investigación en Servicios de Salud en Enfermedades Crónicas (REDISSEC), Barcelona, Spain; 6 Polish Society for Quality Promotion in Healthcare, Krakow, Poland; 7 Department of Public Health, Academic Medical Center, University of Amsterdam, Amsterdam, the Netherlands; 8 Haut Autorité de Santé, Paris, France; 9 Institute for Medical Sociology, Health Services Research and Rehabilitation Science, Faculty of Human Science and Faculty of Medicine, University of Cologne, Cologne, Germany; 10 Department of Epidemiology, Fielding School of Public Health, University of California, Los Angeles (UCLA), Los Angeles, CA, USA; 11 UCLA Center for Health Policy Research, Los Angeles, CA, USA; 12 Department of Public and Occupational Health, EMGO Institute for Health and Care Research, VU University Medical Center, Amsterdam, the Netherlands

**Keywords:** accreditation, certification, health care quality assessment, quality management, patient safety

## Abstract

**Objective:**

To investigate the relationship between ISO 9001 certification, healthcare accreditation and quality management in European hospitals.

**Design:**

A mixed method multi-level cross-sectional design in seven countries. External teams assessed clinical services on the use of quality management systems, illustrated by four clinical pathways.

**Setting and Participants:**

Seventy-three acute care hospitals with a total of 291 services managing acute myocardial infarction (AMI), hip fracture, stroke and obstetric deliveries, in Czech Republic, France, Germany, Poland, Portugal, Spain and Turkey.

**Main Outcome Measure:**

Four composite measures of quality and safety [specialized expertise and responsibility (SER), evidence-based organization of pathways (EBOP), patient safety strategies (PSS) and clinical review (CR)] applied to four pathways.

**Results:**

Accreditation in isolation showed benefits in AMI and stroke more than in deliveries and hip fracture; the greatest significant association was with CR in stroke. Certification in isolation showed little benefit in AMI but had more positive association with the other conditions; greatest significant association was in PSS with stroke. The combination of accreditation and certification showed least benefit in EBOP, but significant benefits in SER (AMI), in PSS (AMI, hip fracture and stroke) and in CR (AMI and stroke).

**Conclusions:**

Accreditation and certification are positively associated with clinical leadership, systems for patient safety and clinical review, but not with clinical practice. Both systems promote structures and processes, which support patient safety and clinical organization but have limited effect on the delivery of evidence-based patient care. Further analysis of DUQuE data will explore the association of certification and accreditation with clinical outcomes.

## Introduction

International Standardization Organization (ISO) certification and healthcare accreditation of provider institutions are widely used as tools for improving or regulating quality and safety in healthcare, and for marketing services across borders. However, there is little hard evidence of the impact of these systems on hospitals to justify the amount of time and money spent on organizational assessment, or to choose between available programmes [1]. Efforts to demonstrate the impact of external assessment have focused almost exclusively on whole hospitals rather than on clinical services.

ISO certification is a voluntary assessment regulated at national, European and international level [2]; healthcare accreditation may be voluntary (at national or international level) or mandatory at governmental level (regional or national). Both systems share the principle of assessment of all departments of the hospital by an external visitor or team against published requirements or standards that focus on systems for quality and safety management more than on resources or results. These standards—or their interpretation—vary within and between countries, especially in accreditation [3]; ISO 9001 is a generic standard for quality management systems in any industry, but accreditation standards are specific to healthcare. Institutions that have been assessed as fully compliant with these standards could be expected to show safer systems than those which are not compliant. Institutions that have in the past been compliant or are currently preparing for assessment could be expected to be partially compliant. Assessment systems vary across Europe [3] but valid standards and reliable assessments should make hospitals safer even if methods are not consistent between individual programmes.

Evidence on the effectiveness of healthcare accreditation based on systematic reviews is limited as few studies have the rigour of randomized controlled trials; evidence of certification has scarcely been researched [4]. The only specific study on the effect of certification in hospitals suggests that, for individual hospitals, higher levels of compliance with quality management standards were more associated with accreditation than with ISO certification, but both systems were significantly better than none [5]. A systematic review [6] classified the impact of accreditation in terms of three types of change: management, organization and culture; professional practice and clinical procedures; and health outcomes. A majority of those studies suggested that accreditation has a positive effect on professional practice, but only a few explored the impact at service (rather than hospital) level where quality management may be considered to be a prerequisite for professional practice.

If hospital-level accreditation or certification is to improve clinical outcomes, it may be expected first to stimulate quality management at the level of care processes. The aim of the present study was to explore whether certification and/or accreditation influences quality management activities at four clinical service levels.

## Methods

### Setting and material

This study was conducted as part of the ‘Deepening our understanding of quality improvement in Europe (DUQuE)’ project, described in detail elsewhere [7]. The main goal of the project was to study the effectiveness of quality improvement systems in European hospitals [8]. We employed a cross-sectional, mixed method and multi-level study design with data collection in the Czech Republic, France, Germany, Poland, Portugal, Spain and Turkey. In each country, we approached 30 randomly selected hospitals (with >120 beds) that handle acute myocardial infection, stroke, hip fracture and delivery. Data were collected with a variety of measures at the hospital-, service- and patient-level. However, data analysed for this paper are based on service-level data collected by on-site audits in a subset of 12 of the 30 hospitals per country, which had consented to in-depth analyses. Data collection took place between May 2011 and February 2012. Details on study recruitment and participation are described elsewhere [7].

Conduct of the on-site data collection was defined in a procedure manual which formed the basis of training of the country coordinators who, in turn, trained their local teams of assessors recruited from existing external assessment programmes. Information obtained by external surveyors during a visit (1.5 days) included: accreditation and certification status of the hospital; quality management activities in clinical services for four conditions—acute myocardial infarction (AMI), deliveries, hip fracture and stroke. These conditions were selected because they cover different hospital areas, occur frequently, are widely researched and provide enough variation in results to allow the analysis.

### Measures of quality management at service level

Data collected on site included structure- and process-related quality management activities in the departments where the four conditions were mainly treated. The individual measures were chosen primarily as markers of compliance with published clinical pathways (reception, triage, diagnostics, intervention, rehabilitation and discharge) together with measures of compliance with WHO guidance on patient safety. Activities were measured with four scales covering the focal areas [10]. Scores for each scale ranged between 0 = ‘no compliance’ and 4 = ‘full compliance’.

- Specialized expertise and responsibility (SER), which reflects clinical leadership and dissemination of clinical guidelines. The SER score was calculated from three items (Table A1)
- Evidence-based organization of pathways (EBOP) refers to critical elements in evidence-based clinical management —5 items for AMI, 7 for stroke and hip fracture and 10 for deliveries (Table A2)
- Patient safety strategies (PSS) measures the use of commonly recommended safety procedures, such as hand hygiene, patient identification and reporting of adverse events. The PSS score was based on 9 items, 11 for delivery (Table A3).
- Clinical review (CR) reflects professional participation in the measurement of clinical practice against formal guidelines. The CR score was based on three items (Table A4)

### Measure of external assessment status

We obtained evidence during the on-site visit whether the hospital was recognized by an established national/regional programme for health service accreditation or certificated for a hospital-wide quality management system under ISO 9001 at the time of data collection.

The rating was based on a five-point Likert scale from ‘0 = No or negligible compliance’ to ‘3 = Extensive compliance/in preparation’ and ‘4 = Full compliance/currently completed’. Compliance was considered with scores 3 or 4. For our analysis, we computed the variable ‘External assessment’ with four categories: (a) Accreditation only, (b) Certification only, (c) Both accreditation and certification, and (d) Neither/none. For example, if a hospital was certified (score = 4) and was in preparation for accreditation (score = 3), then that hospital is in category ‘Both accreditation and certification’ (category = c).

### Hospital characteristics

Hospital characteristics such as ownership (private/public), type (teaching/non-teaching) and the number of beds (<200, 200–500, 501–1000 and >1000 beds) were measured with single items. We additionally stratified for country in our analysis as the data were collected in seven countries.

### Statistical analysis

Appropriate descriptive statistics were calculated to summarize the characteristics for participating hospitals, external assessment and quality management. Dependent variables were measures of quality management at service level: SER, EBOP, PSS and CR. Our independent variable was the measure of external assessment status (accreditation and certification). Further covariates addressed in the models include hospital ownership, teaching status, number of beds and country.

To determine associations, we used a multivariable mixed linear regression model with random intercept by country to account for the clustering of hospitals within country, and adjusted for hospital ownership, teaching status and number of beds. Regression coefficient estimates, standard errors and *P*-values were reported for the relationship between external assessment and the four measures of pathway-level quality management activities separately for each department. The level of statistical significance was set at 5%. All analyses were done in SAS 9.3 (SAS Inc., Cary, NC, USA; 2012).

### Ethical approval

Ethical approval of the study was gained from the Bioethics Committee of the Health Department of the Government of Catalonia in Spain. Approval by national ethical committees was obtained where required.

## Results

### Hospital descriptions

In total, 188 of 210 approached hospitals participated in the DUQuE study. Of these, 74 hospitals participated in the in-depth study. Due to missing data, the final dataset used for these analyses contains 73 hospitals including 291 clinical pathways for patients with AMI, hip fracture, stroke and obstetric deliveries. Most hospitals were public (79%) and nearly half had teaching status (45%); 42% had between 501 and 1000 beds. Of the participating hospitals, 15% were ISO certificated, 34% accredited and 14% had both forms of external assessment (Table 1).

**Table 1:** Distribution of external assessment in sample hospitals (predictor variable)

External assessment	*N*	%
Hospital quality system currently certified by ISO 9001:2000, or in preparation	11	15
Hospital currently accredited, or in preparation	25	34
Hospital certified and accredited	10	14
Hospitals not currently certified or accredited	27	40
All hospitals	73	100

### Measures of quality management at service level

Descriptive results of the four measures of quality management at service level are shown for all four conditions in Table 2. The multi-level regression analysis was performed separately for each (Table 3).

**Table 2:** Descriptive statistics for departmental-level quality management activities (independent variable)

Quality management strategies, mean (SD)	Condition			
	AMI (*N* = 73)	Deliveries (*N* = 72)	Hip fracture (*N* = 73)	Stroke (*N* = 73)
SER	2.7 (1.1)	2.8 (1.1)	2.2 (0.9)	2.7 (1.2)
EBOP	3.2 (0.9)	3.7 (0.3)	2.3 (1.1)	3.0 (1.0)
PSS	2.6 (0.5)	2.7 (0.6)	2.5 (0.5)	2.5 (0.6)
CR	2.1 (1.4)	2.4 (1.4)	1.4 (1.3)	1.9 (1.5)

All measures for quality management at service level were on a range of 0 = no compliance to 4 = full compliance.

**Table 3:** Regression coefficient estimates, standard errors and *P*-values from linear random intercept models for associations between SER, EBOP, PSS, CR and external assessment by condition^a^

External assessment	Quality management at service level											
	SER			EBOP			PSS			CR		
	*b*	SE	*P*-value	*b*	SE	*P*-value	*b*	SE	*P*-value	*b*	SE	*P*-value
AMI												
No accreditation or certification	(reference)			(reference)			(reference)			(reference)		
Accreditation only	0.55	0.35	0.13	0.32	0.32	0.33	0.28	0.18	0.12	0.65	0.43	0.13
Certification only	0.11	0.34	0.75	−0.02	0.34	0.96	0.36	0.21	0.09	−0.10	0.45	0.86
Accreditation and certification	0.92*	0.42*	0.03*	0.23	0.46	0.62	0.52*	0.25*	0.04*	1.35*	0.54*	0.02*
Deliveries												
No accreditation or certification	(reference)			(reference)			(reference)			(reference)		
Accreditation only	0.33	0.41	0.42	−0.03	0.13	0.82	0.25	0.21	0.23	0.75	0.50	0.14
Certification only	0.50	0.40	0.21	0.06	0.12	0.61	0.39	0.24	0.11	0.81	0.50	0.11
Accreditation and certification	0.74	0.47	0.12	−0.06	0.14	0.69	0.41	0.27	0.14	0.81	0.60	0.18
Hip fracture												
No accreditation or certification	(reference)			(reference)			(reference)			(reference)		
Accreditation only	0.14	0.34	0.67	−0.23	0.33	0.49	0.21	0.17	0.22	0.49	0.43	0.26
Certification only	0.13	0.35	0.71	0.24	0.31	0.44	0.21	0.21	0.34	0.29	0.43	0.50
Accreditation and certification	0.52	0.42	0.22	−0.60	0.38	0.12	0.53*	0.24*	0.03*	0.56	0.52	0.29
Stroke												
No accreditation or certification	(reference)			(reference)			(reference)			(reference)		
Accreditation only	0.75	0.40	0.06	0.26	0.33	0.44	0.26	0.17	0.13	1.14*	0.47*	0.02*
Certification only	0.31	0.38	0.42	0.31	0.32	0.34	0.53*	0.22*	0.02*	0.48	0.49	0.34
Accreditation and certification	0.51	0.47	0.29	0.29	0.40	0.47	0.63*	0.24*	0.01*	1.22*	0.59*	0.04*

^a^Multivariable mixed linear regression model with random intercept by country, and adjusted for fixed effects at the hospital level (number of beds, teaching status, public vs. private).

*Significant results.

In the domain of SER (specialized expertise**)**, both accreditation and certification showed a positive association (relative to hospitals with neither system) across all four clinical conditions, especially in hospitals with both systems. Similar associations were shown in patient safety and CR but were notably absent in EBOP where accreditation and certification showed little or no benefit, except in stroke management.

Accreditation in isolation showed benefits in AMI and stroke more than in deliveries and hip fracture; the greatest significant association was with CR in stroke (*b* = 1.14, *P* = 0.02). Certification in isolation showed little benefit in AMI but had a more positive association with the other conditions; the greatest significant association was in PSS with stroke (*b* = 0.53, *P* = 0.02). The combination of accreditation and certification showed least benefit in EBOP, but significant benefits in SER (AMI *b* = 0.92, *P* = 0.03), in PSS (AMI *b* = 0.52, *P* = 0.04; hip fracture *b* = 0.53, *P* = 0.03; stroke *b* = 0.63, *P* = 0.01) and in CR (AMI *b* = 1.35, *P* = 0.02; stroke *b* = 1.22, *P* = 0.04).

## Discussion

The findings suggest that both accreditation and certification are positively associated with measures of clinical leadership (SER), of systems for PSS and of CR, but not with critical elements of EBOP. Both systems appear to promote structures and processes that support patient safety and clinical organization but have limited effect on the delivery of evidence-based practice.

Most of the measures of specialist management, evidence-based organization and CR were based on quality standards and audit tools recommended by NICE in England and SIGN in Scotland; these include measures both of good clinical practice (which can be implemented in individual hospital departments) and of service configuration (which require coordination between departments and between hospitals). In some countries, particularly the UK, the introduction of fast-track pathways and specialized units has become a priority for service re-engineering in recent years, especially for the management of AMI, stroke and hip fracture, which are characterized by unplanned, emergency admissions demanding rapid access to new technologies and skills. In contrast, maternity care has been the focus of research, evaluation and standardization for hundreds of years; there is less opportunity for developing innovative clinical pathways or systems, or for making improvements driven by accreditation or certification. Moreover, the definitions used in this study excluded complex obstetrics. Most of the measures of PSS were based on guidance from the WHO global challenge for patient safety. Their uptake is associated both with accreditation and certification.

Comparing the differences in the size of associated effects between accreditation and certification shows no clear pattern. For clinical leadership, accreditation showed greater impact than certification in AMI (*b* = 0.55 versus 0.11) and stroke (*b* = 0.75 versus 0.31), but the reverse in deliveries (*b* = 0.33 versus 0.50). Accreditation also appeared to confer advantage over certification in CR (AMI *b* = 0.65 versus −0.10, hip fracture *b* = 0.49 versus 0.29 and stroke *b* = 1.14 versus 0.48). Accreditation was more weakly associated with patient safety systems than was certification in AMI (*b* = 0.28 versus 0.36), deliveries (*b* = 0.25 versus 0.39) and stroke (*b* = 0.26 versus 0.53).

Accreditation appears to have an overall advantage over certification in clinical leadership and review but few of these results are statistically significant. Where both systems, on their own, show a positive association with quality management, their effect in combination appears to be greater and more significant. A paradox appears with CR in AMI; accreditation alone shows a modest positive effect (*b* = 0.65) and certification alone a weak negative effect (*b* = −0.10), but hospitals with both systems show a strong positive effect (*b* = 1.35, *P* = 0.02) compared with hospitals having neither system.

### Interpretation and relation with other studies

If external assessment has any effect on hospitals, we could expect that any system would have more impact than no system and that accreditation standards specific to healthcare would have a greater impact on patient care than an ISO standard for quality management in general industry. Many of the measures used in this study to assess PSS, CR and clinical leadership are implicit or explicit in the standards used for healthcare accreditation, but not in ISO 9001. Several ‘interpretation documents’ have been developed to guide the use of ISO in healthcare but this study did not explore which, if any were used in certification of the sample hospitals.

The MARQuIS study of 89 hospitals in different European countries suggested that hospitals, which were either accredited or ISO certified, scored higher on quality and safety process and outcomes than hospitals that had neither form of external assessment [6]. It also implied that hospitals that were accredited scored higher on composite measures of quality and safety than hospitals that are ISO certified. Overall, the current study is partly consistent with these conclusions but the associations are variable between focal areas and between clinical services.

In the current study, the combination of accreditation and certification was a more powerful predictor of quality management at service level than either assessment in isolation. This may suggest that both accreditation and certification have exclusive beneficial features which they do not share or that hospitals which voluntarily join both systems are a self-selected group of high achievers. Furthermore, we have found some clustering for accreditation in two countries and for the combination of assessment in another country.

Finally, the DUQuE project also collected data on other forms of external assessment, including regulatory supervision and accreditation of training. Similar analysis of these to identify associations with departmental organization and clinical outcome might help to put the contribution of certification and accreditation into context. Until the interactions between various types of external assessment, and their effects on clinical outcomes, are better understood, we should beware studying the impact of accreditation in isolation.

### Limitations

Common limitations of the DUQuE project are described elsewhere [8]. Given the variation in standards and assessment processes used in different countries, the impact of accreditation is likely to be equally inconsistent between countries; the inclusion of national or regional programmes and exclusion of international programmes adds further heterogeneity. Similarly, although ISO 9001 is international, its interpretation in healthcare is inconsistent between auditors, despite the development of guidance documents to translate it into the hospital setting. In the regulated system of the ISO, certification bodies must themselves be ‘accredited’ by an authorized national accreditation service. Healthcare accreditation organizations are not similarly regulated, but they may be accredited by the International Accreditation Program of the International Society for Quality in Health Care. Neither certification nor accreditation should be regarded as standardized across borders, nor expected to have a consistent impact on hospitals and patient care.

## Conclusion

Even allowing for the uncertainty of small numbers, this study does show that some elements of quality management at the clinical level are associated with certification and/or accreditation, but others are not. Neither type of assessment in isolation had a consistent and significant impact on clinical services, but hospitals that were both certified and accredited scored significantly higher in services managing stroke and AMI, especially in relation to CR and patient safety. Further enquiry into these results could usefully probe the possibility of self-selection by hospitals intent on demonstrating their excellence, confounding variation in assessment standards and procedures across national borders, and the association of certification and accreditation with individual components of the composite measures of organization and outcome. The association of external assessment with clinical outcome will be explored in a later paper from the DUQuE project.

## Funding

This study is funded by the European Commission's Seventh Framework Programme FP7/2007-2013 under grant agreement no. 24188. Funding to pay the Open Access publication charges for this article was provided by European Community's Seventh Framework Programme (FP7/2007–2013) under grant agreement no 241822.

## Appendix 1

### The DUQuE Project Consortium comprises

Klazinga N, Kringos DS, Lombarts K and Plochg T (Academic Medical Centre-AMC, THE NETHERLANDS); Lopez MA, Secanell M, Sunol R and Vallejo P (Avedis Donabedian University Institute-Universitat Autónoma de Barcelona FAD. Red de investigación en servicios de salud en enfermedades crónicas REDISSEC, SPAIN); Bartels P (Central Denmark Region & The Department of Clinical Medicine, Aalborg University, Aalborg, DENMARK) Kristensen S (Central Denmark Region & Center for Healthcare Improvements, Aalborg University, Aalborg, DENMARK); Michel P and Saillour-Glenisson F (Comité de la Coordination de l'Evaluation Clinique et de la Qualité en Aquitaine, FRANCE); Vlcek F (Czech Accreditation Committee, CZECH REPUBLIC); Car M, Jones S and Klaus E (Dr Foster Intelligence-DFI, UK); Garel P and Hanslik K (European Hospital and Healthcare Federation-HOPE, BELGIUM); Saluvan M (Hacettepe University, TURKEY); Bruneau C and Depaigne-Loth A (Haute Autorité de la Santé-HAS, FRANCE); Shaw C (Independent Consultant, UK); Hammer A, Ommen O and Pfaff H (Institute for Medical Sociology, Health Services Research and Rehabilitation Science, University of Cologne-IMVR, GERMANY); Groene O (London School of Hygiene and Tropical Medicine, UK); Botje D and Wagner C (The Netherlands Institute for Health Services Research-NIVEL, THE NETHERLANDS); Kutaj-Wasikowska H and Kutryba B (Polish Society for Quality Promotion in Health Care-TPJ, POLAND); Escoval A (Portuguese Association for Hospital Development-APDH, PORTUGAL) and Franca M (Portuguese Society for Quality in Health Care-SPQS, PORTUGAL); Almeman F, Kus H and Ozturk K (Turkish Society for Quality Management in Healthcare-SKID, TURKEY); Mannion R (University of Birmingham, UK); Arah OA, Chow A, DerSarkissian M, Thompson C and Wang A (Palo Alto Medical Foundation Research Institute, Palo Alto, California, USA); Thompson A (University of Edinburgh, UK)

## Appendix 2

(Tables A1–A4)

**Table A1:** Items of SER of each pathway

	AMI	Stroke	Hip fracture	Delivered
There is a strategic group within the hospital responsible for the overall clinical management	x	x	x	x
There are clinical leaders with specialist training who are formally recognized as having principal responsibility for the overall clinical care	x	x	x	x
Evidence-based clinical guidelines have been formally adopted and disseminated by the clinical staff for the management of patients	x	x	x	x

**Table A2:** Items of EBOP of each pathway

	AMI	Stroke	Hip fracture	Deliveries
There are written criteria and procedures for fast-track admission and treatment of patients presenting with acute chest pain	x			
Arrangements ensure that eligible STEMI (S-T elevation myocardial infarction) patients can receive thrombolysis within 30 min after arrival at the hospital	x			
Immediate access is available at all times (24/7) to a specialist physician to determine whether coronary revascularization is appropriate	x			
Facilities are immediately available for performance and transport for emergency coronary angiography	x			
Facilities are immediately available for performance and transport for percutaneous coronary intervention	x			
There is an agreed procedure for appropriate patients directly be transport for ambulance personnel to a stroke unit		x		
Agreed procedures ensure that patients with suspected stroke are assessed for thrombolysis receiving, if clinically indicated		x		
A thrombolysis service is available 7 days a week in the hospital or by formal arrangement elsewhere		x		
Agreed procedures ensure that patients with acute stroke have their swallowing screened be a specially trained healthcare professional		x		
Protocols and procedures are available in order for patients to receive brain imaging within 1 h after arrival at the hospital		x		
Protocols are in place to ensure documented multidisciplinary goals are agreed within 5 days after admission to the hospital		x		
There is immediate access (1 h) to a specialist acute stroke unit (or area) for those with persisting neurological symptoms		x		
The guidelines require that medical staff assess patients suspected of having a fractured hip within 1 hour after arrival in the ED (or of the incident if already in the hospital)			x	
The guidelines require a multidisciplinary assessment plan and individual goals for rehabilitation to be documented within 24 h post-operatively			x	
Magnetic resonance imaging is immediately available if hip fracture is suspected despite negative plain X-rays			x	
The guideline requires that all patients presenting with a fragility (pathological) fracture are managed on a ward with routine access to acute orthogeriatric medical support			x	
Whenever clinically appropriate, surgery is performed within 48 h after admission			x	
Guidelines require that all patients undergoing hip fracture surgery receive antibiotic prophylaxis			x	
Guidelines require that, if the patient's overall medical condition allows, mobilization begins within 24 h post-operatively			x	
A structured, accurate record of all events during the antenatal, childbirth and postnatal periods is maintained for every woman and child				x
All women, who have epidural analgesia or an operative delivery, have their pain assessed using a pain assessment tool approved by the hospital				x
There is prompt access to ultrasound facilities with trained staff				x
There is a procedure that guarantees that all women who are identified in the screening programme as at risk of rhesus disease are properly managed				x
Each woman receives one-to-one midwifery care during established labour and childbirth by a trained midwife				x
Epidural analgesia is available at all times				x
Adult intensive care facilities and specialist medical backup are available on-site				x
Patient monitoring equipment and clinical expertise in its management are available within the obstetric unit				x
There is a system in place to ensure that anaesthetic and theatre services respond within 30 min to obstetric emergencies and expedite delivery in the event of maternal or foetal compromise				x
All babies are clinically examined prior to discharge from hospital and/or within 72 h of birth, by a suitable qualified healthcare professional				x

**Table A3:** Items of PSS of each pathway

	AMI	Stroke	Hip fracture	Deliveries
Patients are identified by bracelet	x	x	x	x
Safety boxes for disposal of injection devices are available in sufficient quantities for the number of injections administered	x	x	x	x
Promotional hand hygiene reminders are on display in the workplace	x	x	x	x
Staff are provided with a readily accessible alcohol-based hand rub at the point of patient care	x	x	x	x
There is no concentrated potassium chloride (KCl) stored in patient service areas	x	x	x	x
Diagrammatic instructions for resuscitation are available in resuscitation areas	x	x	x	x
Each emergency ‘crash cart’ has a completed checklist of equipment and supplies	x	x	x	x
There is a system to report adverse events to patients	x	x	x	x
During 2010, CR included analysis of reported adverse events	x	x	x	x

**Table A4:** Items of CR of each pathway

	AMI	Stroke	Hip fracture	Deliveries
During 2010, CR included analysis of routine clinical indicators on the management of the condition	x	x	x	x
There is a multidisciplinary audit/review of practice against the guidelines	x	x	x	x
Professionals participate or have direct feedback on results of audit/review of practice against guidelines	x	x	x	x
